# A Strategy to Design Cu_2_MoS_4_@MXene Composite With High Photothermal Conversion Efficiency Based on Electron Transfer Regulatory Effect

**DOI:** 10.3389/fbioe.2022.902312

**Published:** 2022-05-13

**Authors:** Lihua Li, Jifan Zhang, Yang Lin, Yongfeng Zhang, Shujie Li, Yanzhen Liu, Yingxu Zhang, Leilei Shi, Shouzhang Yuan, Lihao Guo

**Affiliations:** ^1^ NORINCO Kunming Institute of Physics, Kunming, China; ^2^ The Eighth Affiliated Hospital, Sun Yat-Sen University, Shenzhen, China; ^3^ School of Advanced Materials and Nanotechnology, Interdisciplinary Research Center of Smart Sensors, Xidian University, Xi’an, China

**Keywords:** photothermal conversion, MXene, heterostructure, nanocomposite, electron migration

## Abstract

Using photothermal therapy to treat cancer has become an effective method, and the design of photothermal agents determines their performance. However, due to the major radiative recombination of a photogenerated electron in photothermal materials, the photothermal performance is weak which hinders their applications. In order to solve this issue, preventing radiative recombination and accelerating nonradiative recombination, which can generate heat, has been proved as a reasonable way. We demonstrated a Cu_2_MoS_4_@MXene nanocomposite with an obviously enhanced photothermal conversion efficiency (*η* = 87.98%), and this improvement can be attributed to the electron migration. Then, a mechanism is proposed based on the electron transfer regulatory effect and the localized surface plasmon resonance effect, which synergistically promote nonradiative recombination and generate more heat. Overall, our design strategy shows a way to improve the photothermal performance of Cu_2_MoS_4_, and this method can be extended to other photothermal agents to let them be more efficient in treating cancer.

## Introduction

In the 21st century, the cancer problem has become more dominant and malignant, which is one of the most threatening public health questions and causes over 9.6 million deaths annually in the world (Massagué and Obenauf, 2016; Steeg, 2016). However, there is still a method that can rapidly and completely treat cancer because of metastasis; meanwhile, the traditional treatments, for e.g., radiotherapy and chemotherapy, may induce severe side effects which are traumatic for patients (Yilmaz et al., 2007). In recent decades, the optical treatment has been considered an efficient and less traumatic approach to treat primary and metastatic tumors, and the photothermal therapy (PTT) has been synergistically used with traditional methods and has shown a satisfied treatment effect (Chang et al., 2019; Yuan et al., 2020). In brief, under optical irradiation, photothermal reagents can generate localized hyperthermia and treat cancer. However, most of the existing photothermal materials lack photothermal performance due to the minority nonradiative recombination of the photogenerated electron. The low photothermal conversion determines that more materials or higher laser power will be used to achieve the heat temperature, which can easily cause damage to patients (Zhou et al., 2018; Xi et al., 2020). So, the suitable photothermal materials with high photothermal performance are urgently needed.

Ternary chalcogenide, Cu_2_MoS_4,_ is a representative material that has been exploited in photothermal therapy (Chen et al., 2014; Chang et al., 2019). However, the photothermal performance of Cu_2_MoS_4_ is weak which is common for simplex phototherapy reagents and can be explained by the band theory that the photothermal effect of Cu_2_MoS_4_ is mainly induced by the nonradiative recombination (producing phonon) of photogenerated electron–hole pairs, but this recombination is low compared with the radiative recombination (Zhang et al., 2015; Cheng et al., 2018; Zhang et al., 2018; Lv et al., 2021). Hence, it is highly desirable to improve the photothermal performance of Cu_2_MoS_4_ to make it a suitable photothermal reagent that achieves better anticancer outcomes.

In order to accelerate the probability of nonradiative recombination in the electron transfer process and prevent radiative recombination, which can efficiently enhance the photothermal effect of Cu_2_MoS_4_, constructing a heterostructure of noble metal or graphene and Cu_2_MoS_4_ has been proved as a reasonable method (Zhang et al., 2016; Chang et al., 2020). The high conductivity can lead the electron to migrate from Cu_2_MoS_4_ to noble metal or graphene when they come into contact with Cu_2_MoS_4_, and then the radiative recombination can be prevented while the probability of nonradiative recombination increases, thus enhancing photothermal performance (Rameshbabu et al., 2017). Nevertheless, the composite process of Cu_2_MoS_4_ and noble metal (usually nanoparticles) or graphene is difficult, and the interface resistance of metal nanoparticles may hinder the transfer of electrons, weakening the migration. Hence, MXene, a new member of 2D materials considered 2D transition metal carbides or nitrides with metallic conductivity, has become a substitute for noble metal and graphene, and the abundant surface termination groups on MXene’s surface provide a large number of sites for Cu_2_MoS_4_ to anchor on (Guo et al., 2021; Mohammadi et al., 2021; Qiu et al., 2021). Meanwhile, owing to the high work function, superior electron conductivity, and lower interface resistance (compared with noble metal nanoparticles) of MXene, the caused electron transfer regulatory effect can enhance the photothermal performance of composites, which is similar to the aforementioned noble metal (Mariano et al., 2016; Chang et al., 2020; Li et al., 2021). More interestingly, under visible light radiation at 800 nm (1.5 eV), the MXene can produce the localized surface plasmon resonance (LSPR) effect which is a novel method to assist the separation of electrons and further stimulate the generation of heat (Mariano et al., 2016; Demellawi et al., 2018; Lioi et al., 2019).

**SCHEME 1:**
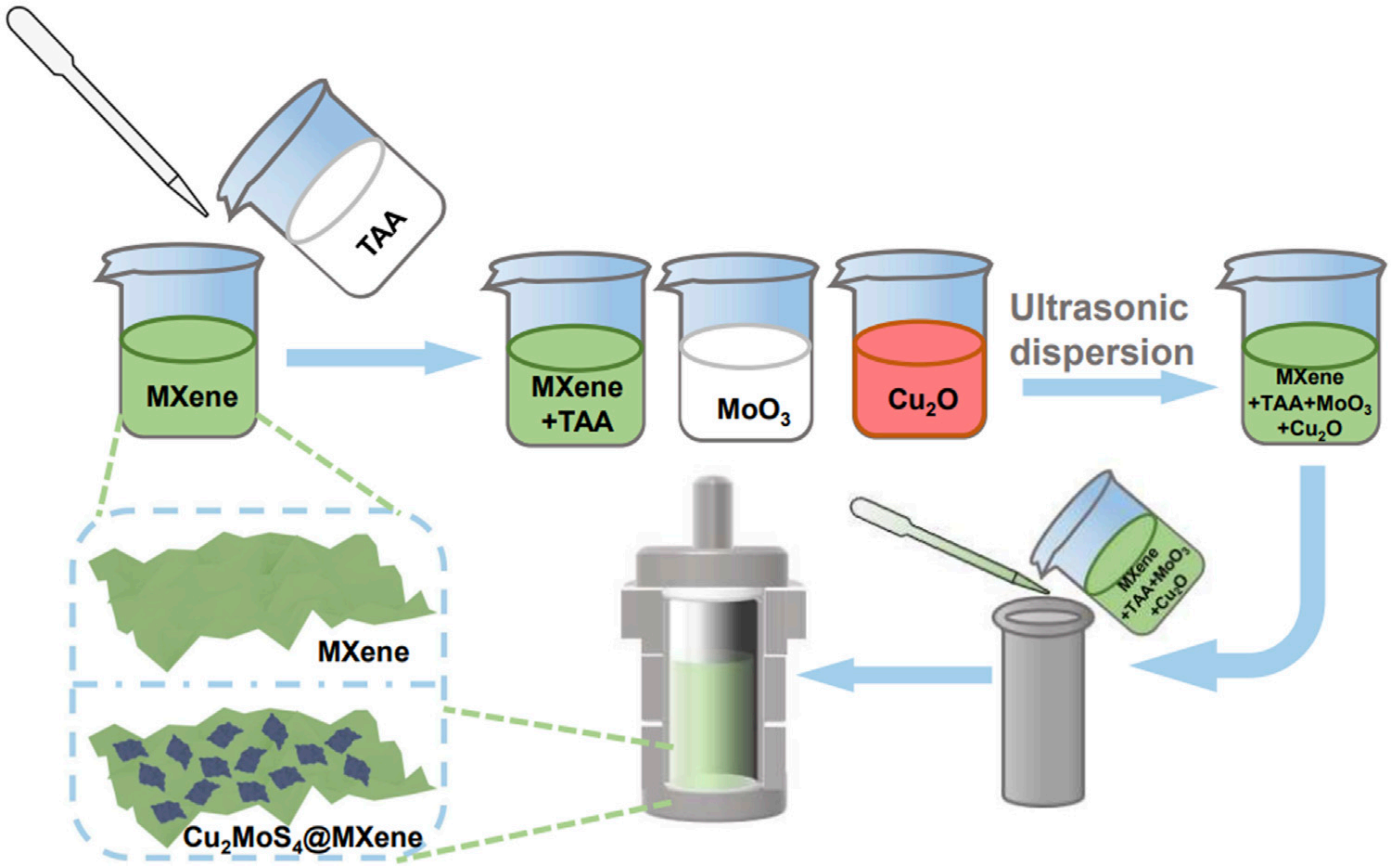
Process of synthesizing Cu_2_MoS_4_@MXene nanocomposites.

We introduced Ti_3_C_2_T_x_ MXene nanosheets to improve the photothermal performance of Cu_2_MoS_4_, and a Cu_2_MoS_4_@MXene nanocomposite was synthesized (Scheme 1). The results of the photothermal conversion experience confirmed our hypothesis, and the best performance of the nanocomposite obtained can increase the temperature by more than 55 °C under NIR radiation (1.0 W/cm^−2^ at 808 nm) with an obviously enhanced photothermal conversion efficiency (87.98%) compared with pure Cu_2_MoS_4_ (72.07%). Using the absorption spectrum and photoluminescence (PL) spectrum, the electron transfer process can be verified, and the obvious quenching phenomenon, i.e., weakened radiative recombination, reflects the rapid separation and transfer of photogenerated electrons. Based on the experimental results and band theory, we proposed a mechanism of enhanced photothermal performance that is mainly caused by promoted separation and transfer of electron and nonradiative recombination, owing to the electron transfer regulatory effect and LSPR effect. Therefore, these results suggest that the Cu_2_MoS_4_@MXene nanocomposite with enhanced photothermal performance could be used to treat cancers.

## Experimental Section

### Preparation of Ti_3_C_2_T_x_ MXene Nanosheets

Using a minimally intensive layer delamination (MILD) method as previously reported (Halim et al., 2014), the Ti_3_C_2_T_x_ MXene nanosheets were prepared from commercial Ti_3_AlC_2_ MAX purchased from Forsman Scientific Co. In detail, first, the etching agent was prepared by gently adding 1.5 g LiF into 20 ml of 9 M HCl with continuous stirring. After LiF was totally dissolved, 1 g Ti_3_AlC_2_ MAX powder was slowly added to the etching agent, and the mixture was continuously stirred at 35°C for 30 h. Afterward, the product was washed several times with deionized water (DI water), and when the pH of the supernatant reached 6, the supernatant was ultrasonicated for 1.5 h with N_2_ atmosphere protection in an ice bath. Then, the Ti_3_C_2_T_x_ MXene nanosheets were obtained after centrifugation (3,500 rpm for 1 h).

### Preparation of Cu_2_O Precursor

The Cu_2_O precursor was synthesized by reducing copper hydroxide. NaOH (30 ml 3.75 M) solution was added dropwise in CuSO_4_ (30 ml 0.5 M) solution with continuous stirring to prepare Cu(OH)_2_ colloid; meanwhile, glucose (C_6_H_12_O_6_, 30 ml 0.75 M) solution was prepared and kept at 60°C. Then, the Cu(OH)_2_ colloid was heated to 60°C and added into glucose solution drop by drop at 60°C by placing in a water bath. Then, the mixture color gradually turned brick-red which indicates the successful preparation of Cu_2_O. After allowing the colloid to react at 60°C for 30 min, the precursor was obtained through filtrating colloid and vacuum drying.

### Synthesis of Cu_2_MoS_4_@MXene Nanocomposites

To synthesize Cu_2_MoS_4_@MXene nanocomposites, 0.02 g MXene was first dissolved in 30 ml DI water, and (0 g, 0.14 g, 0.42 g, 0.70 g, 0.98 g, and 1.3 g) thioacetamide (TAA) was added into MXene colloidal solution. Meanwhile, (0 g, 0.17 g, 0.51 g, 0.85 g, 1.2 g, and 1.6 g) MoO_3_ and (0 g, 0.12 g, 0.36 g, 0.60 g, 0.84 g, and 1.1 g) Cu_2_O were ultrasonically dispersed into 5 ml DI water, respectively. Then, three dispersion solutions were transferred into a 100-ml tailor-made Teflon reactor, and using a microwave, the reaction temperature could be rapidly elevated to 150°C within 3 min. Afterward, the reaction temperature was maintained at 150°C for 2 h, and the Cu_2_MoS_4_@MXene nanocomposite (marked Cu_2_MoS_4_, Cu_2_MoS_4_@MXene-1, Cu_2_MoS_4_@MXene-3, Cu_2_MoS_4_@MXene-5, Cu_2_MoS_4_@MXene-7, and Cu_2_MoS_4_@MXene-9 for different ratios) products were obtained after washing and drying.

### Photothermal Effect of Cu_2_MoS_4_@MXene Nanocomposites

In the following photothermal performance experiment, a series of concentration (0, 50, 100, 200, 500, and 1,000 μg/ml) solutions (1 ml) of six samples were prepared in an Eppendorf tube, respectively, and a NIR laser (1.0 W/cm^2^) at 808 nm was used to irradiate the samples (Zhang et al., 2021). Then, during 600 s of irradiation, the temperature change of samples was monitored using an infrared thermal imaging camera and recorded on a computer connected to the camera in real-time. The photostability was tested (500 μg/ml) by repeating the heating (laser on for 600 s)/cooling (laser off for 600 s) processes three times (power density is 1.0 W/cm^2^). Furthermore, the laser was modulated for 0.5 W/cm^2^, 1.0 W/cm^2^, and 1.5 W/cm^2^ to evaluate the influence of power density.

### Calculation of Photothermal Conversion Efficiency

Based on the results of the photothermal experiment, the photothermal conversion efficiency can be calculated according to Equation 1:

$$\eta =\frac{hS\left(T_{max}-T_{0}\right)-Q_{dis}}{W\left(1-10^{-A_{808}}\right)},$$ (1)

where η is the photothermal conversion efficiency, h (Wcm^−2^ K^−1^) is the heat transfer coefficient, S (cm^2^) is the surface area of quartz cuvette, T_max_ (K) is the highest equilibrium temperature, T_0_ (K) is the surrounding temperature, Q_dis_ (W) is the heat loss which is approximate to 0, W is the power density of the laser, and A_808_ is the absorbance of samples at 808 nm. Moreover, the T_max_, T_0_, and A_808_ can be measured, while the hS is calculated through Equation 2:

$$hS=\frac{m_{W}C_{W}}{\tau_{s}},$$ (2)

where m_W_ and C_W_ represent the total mass and the specific heat capacity of solvent and water, respectively, and τ_s_ is the time constant which can be obtained through Equation 3:

$$t=-\tau_{s}\times ln\theta =-\tau_{s}ln\frac{T-T_{0}}{T_{max}-T_{0}},$$ (3)

Using the recording temperature T, the τ_s_ can be calculated, and then the photothermal conversion efficiency is calculated (Chen et al., 2019).

### Characterization and Measurement

The morphologies and compositions of precursors and composites were characterized using the transmission electron microscope (TEM, JEOL-2100F), the X-ray diffraction spectrometer (XRD, Bruker D8 Advance), Raman and photoluminescence (PL) spectroscopy (Renishaw inVia), and UV-vis-NIR spectra (JASCO V-570). Infrared thermal imaging was monitored using an IR thermal camera (TELEDYNE FLIR Exx) and recorded using a computed connected to the camera.

## Result and Discussion

### Characterization of Precursors and Cu_2_MoS_4_@MXene Nanocomposites

As illustrated in Scheme 1, we have prepared Cu_2_MoS_4_@MXene nanocomposites through the *in situ* hydrothermal method (see details in Experimental Section). In brief, the prepared MXene nanosheets were first mixed with thioacetamide (TAA) in water; meanwhile, the MoO_3_ and Cu_2_O were ultrasonically dispersed in water. Then, the reaction fluid prepared by mixing three dispersions was poured into a microwave hydrothermal reactor and allowed to react using a microwave. After washing with DI water, the Cu_2_MoS_4_@MXene nanocomposites can be obtained. More importantly, this process allows Cu_2_MoS_4_ nanoplates to uniformly grow on MXene nanosheets which cannot be reached by the physical mixing method. Furthermore, due to the high quality of Cu_2_MoS_4_@MXene nanocomposites and the unique effect caused by composite processes, such as band engineering and high electron conductivity of MXene, a more excellent photothermal conversion performance can be achieved than than that of pure Cu_2_MoS_4_ nanoplates.

**FIGURE 1:**
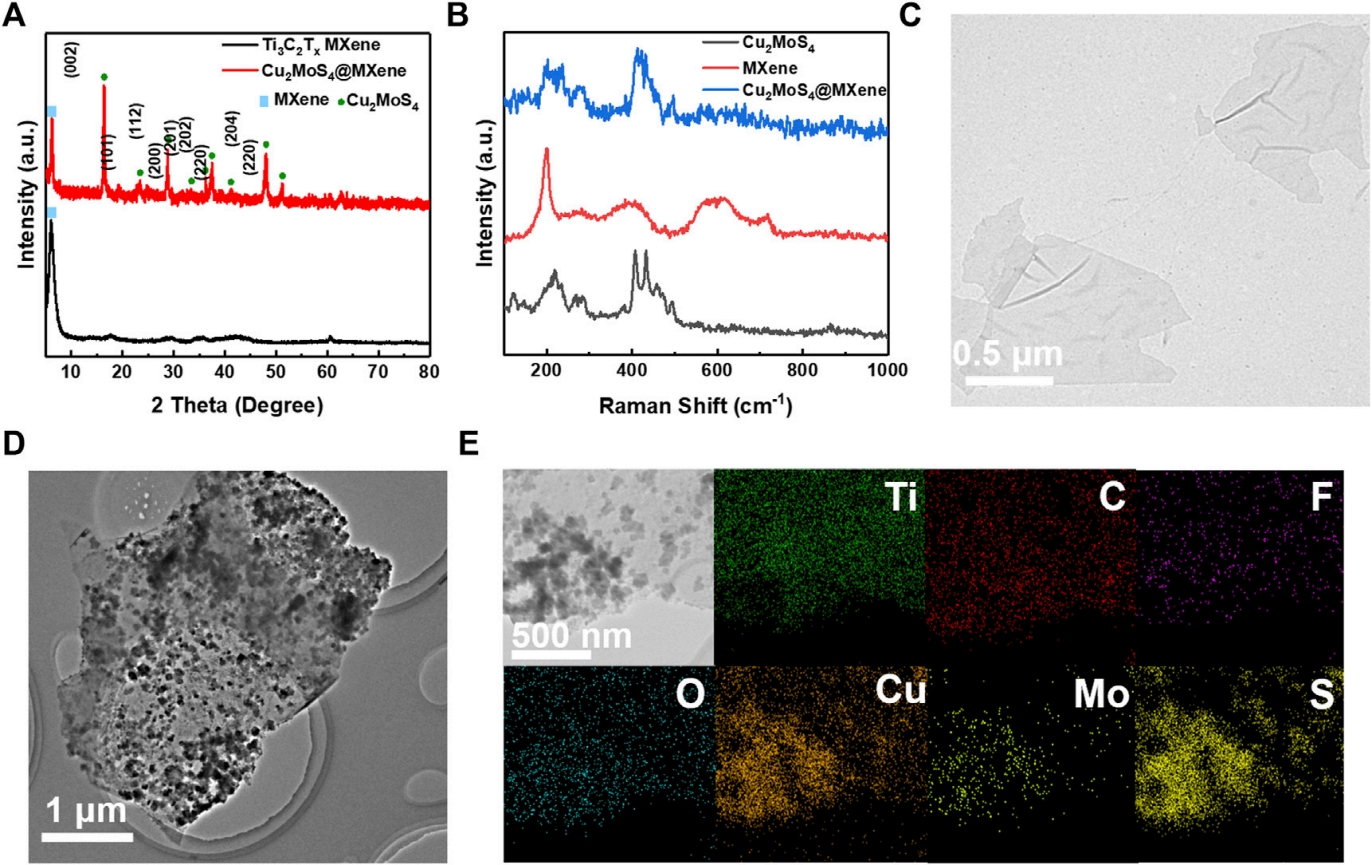
Characterization of precursor and Cu_2_MoS_4_@MXene nanocomposites. **(A)** XRD pattern of Ti_3_C_2_T_x_ MXene and Cu_2_MoS_4_@MXene. **(B)** Raman spectra of Cu_2_MoS_4_, MXene, and Cu_2_MoS_4_@MXene. TEM images of **(C)** MXene nanosheets and **(D)** Cu_2_MoS_4_@MXene. **(E)** EDS mapping of Ti, C, F, O, Cu, Mo, and S of Cu_2_MoS_4_@MXene.

As for the basics of nanocomposites, the MXene nanosheets were first prepared using the MILD method (Halim et al., 2014). The Ti_3_AlC_2_ (MAX) precursor was etched with LiF and HCl. During the etching process, the Al layer in the MAX phase was selectively etched and Ti_3_C_2_T_x_ nanosheets remained. As shown in the XRD pattern (Figure 1A), both the diffraction peaks at 39°, which can be indexed to the (104) plane of Ti_3_AlC_2_ MAX, disappear, and the (002) peak left shifts to 6.5°, indicating successful preparation of Ti_3_C_2_T_x_ nanosheets (Lipatov et al., 2016). According to the pattern of the Raman spectrum (Figure 1B), the vibration modes of MXene nanosheets can be divided into two types: out-of-plane mode (A_1g_) and in-plane mode (E_g_), and the vibration peak at 198 cm^−1^, 715 cm^−1^ and 253 cm^−1^, and 502 cm^−1^ can be well assigned to these two modes, respectively (Hu et al., 2015). Using TEM, the monolayer Ti_3_C_2_T_x_ nanosheets can be seen with a size of approximately 1 μm, as shown in Figure 1C. The results of these characterization methods show that the high-quality MXene nanosheets have been successfully produced and can be used in the next composite process.

**FIGURE 2:**
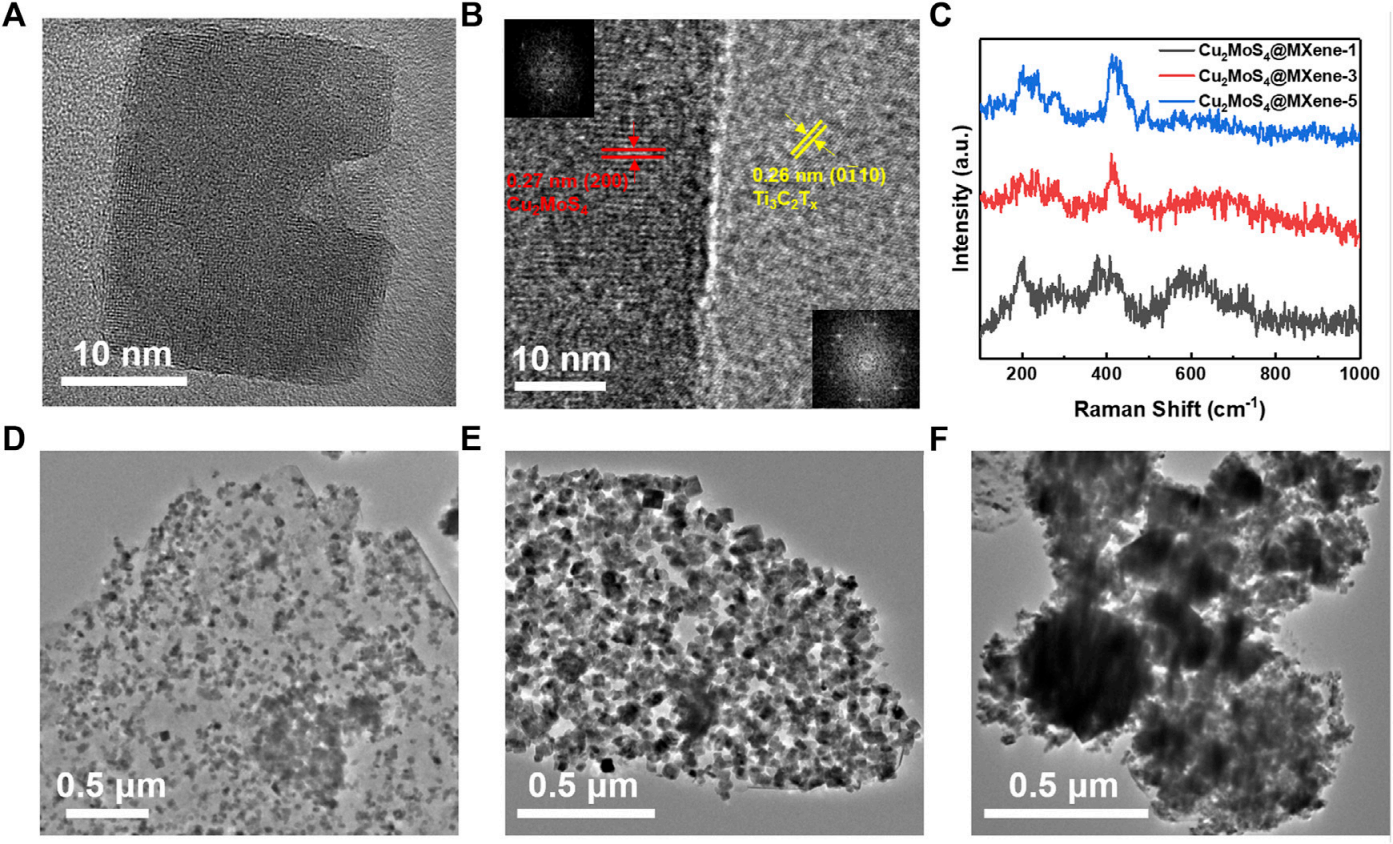
Characterization of different Cu_2_MoS_4_@MXene nanocomposites. **(A,B)** HRTEM of Cu_2_MoS_4_@MXene (inset in B is the FFT of Cu_2_MoS_4_ and MXene, respectively). **(C)** Raman spectra of Cu_2_MoS_4_@MXene-1, Cu_2_MoS_4_@MXene-3, and Cu_2_MoS_4_@MXene-5. **(D–F)** TEM images of Cu_2_MoS_4_@MXene-1, Cu_2_MoS_4_@MXene-5, and Cu_2_MoS_4_@MXene-9, respectively.

Because of many hydrophilic terminations planted on the surface of MXene nanosheets during the liquid etching process, the MXene can be well dispersed in water, as shown in Supplementary Figure S1, which ensures the stability of MXene solution in the hydrothermal process. After the hydrothermal process, the morphology of MXene nanosheets has undergone an obvious change (Figure 1D), and there are many nanoplates anchored on the surface of unimpaired MXene nanosheets. In Figure 1E, the distribution of elements reflected by EDS shows that the MXene is still intact, and the Cu_2_MoS_4_ nanoplates are only synthesized on the surface of MXene nanosheets which meets our expectations. Interestingly, it can be seen that the size of Cu_2_MoS_4_@MXene nanocomposites appears bigger than that of pure MXene nanosheets, and this phenomenon may be a combination of several nanosheets, which is caused by some anchored Cu_2_MoS_4_ that can connect adjacent MXene nanosheets. In order to further confirm the successful synthesis of Cu_2_MoS_4_@MXene nanocomposites, the XRD and Raman spectra were used. In Figure 1A, the XRD result of Cu_2_MoS_4_@MXene nanocomposites illustrates that the Cu_2_MoS_4_ on the MXene nanosheets has good crystallinity, and the major peaks at 6.1°, 16.3°, 28.8°，37.4°, and 47.9° are well indexed to the MXene and tetragonal-phase of Cu_2_MoS_4_ (*P* $\bar{4}$ *2m*, JCPDS 81–1,159) (Lipatov et al., 2016; Zhang K. et al., 2017). Moreover, no evident diffraction peak of TiO_2_ is consistent with the aforementioned verdict that the MXene nanosheets are unimpaired during the hydrothermal process which is because of a reductive environment made by the hydrolytic process of TAA. This conclusion can also be proved by the Raman spectrum in Figure 1B, where there is no peak at 150 cm^−1^ assigned to TiO_2_ (Zhang C. J. et al., 2017). In addition to this, the peaks of Cu_2_MoS_4_@MXene nanocomposites at 200 cm^−1^, 233 cm^−1^, and 415 cm^−1^ represent the coexistence of Cu_2_MoS_4_ and MXene (Kim et al., 2017). In the microscopic image, the size of tetragonal Cu_2_MoS_4_ nanoplates that anchored on the MXene surface is about 20 nm (Figure 2A). Furthermore, the high-resolution TEM image in Figure 2B shows the crystal lattice spacing between 0.27 and 0.26 nm, which can be ascribed to the (200) plane of Cu_2_MoS_4_ and the (0,110) plane of MXene, and the FFT images (inset of Figure 2B) illustrate the tetragonal and hexagonal structure of Cu_2_MoS_4_ and MXene, respectively (Zhang K. et al., 2017; Agresti et al., 2019). Thus, according to the spectrum analysis and microscopic analysis, it can be confirmed that the Cu_2_MoS_4_@MXene nanocomposites have been successfully prepared with high quality.

Considering the ratio of Cu_2_MoS_4_ and MXene in Cu_2_MoS_4_@MXene nanocomposites may affect their performance, we prepared a series of nanocomposite samples with a gradient ratio. There is a regular variation in the Raman spectrum (Figure 2C and Supplementary Figure S2), and with the increased ratio of Cu_2_MoS_4_, the vibration peak of MXene becomes decrescent and that of Cu_2_MoS_4_ becomes stronger. Moreover, the microscopic morphology of Cu_2_MoS_4_@MXene-1, Cu_2_MoS_4_@MXene-5, and Cu_2_MoS_4_@MXene-9 prove the result of the Raman spectrum, which shows that the observed coverage ratio of anchored Cu_2_MoS_4_ nanoplates on MXene nanosheets increases, but when the ratio of Cu_2_MoS_4_ is too high (Cu_2_MoS_4_@MXene-9), the excessive aggregation occurs which is unfavorable in application (Fang et al., 2020). In addition, when the ratio of Cu_2_MoS_4_ becomes high, there are more evident signals of Cu and Mo elements in EDS mapping (Supplementary Figure S3) compared with the low ratio sample. Also, an evident aggregation of Cu_2_MoS_4_ can be seen, which further verified the aforementioned point. However, even if the ratio is excessively increased, there are still no Cu_2_MoS_4_ nanoplates lying outside the MXene nanosheets because of the anchoring effect mentioned earlier, which ensures the contact of Cu_2_MoS_4_ with high conductivity MXene and the electron transfer regulatory effect.

### Optical Properties of Cu_2_MoS_4_@MXene Nanocomposites

As introduced earlier, the MXene can affect the photothermal performance of nanocomposites through band engineering and regulatory effect, so to make clear the role of MXene in this process and whether the nanocomposite will gather an improved performance, the following optical properties of nanocomposites are enumerated: the result of the PL spectrum suggests that the nanocomposites’ PL intensity is different from pure Cu_2_MoS_4_, and the samples marked as Cu_2_MoS_4_@MXene-1, Cu_2_MoS_4_@MXene-3, and Cu_2_MoS_4_@MXene-5 have a lower intensity. According to the band theory, the PL intensity is associated with the recombination of photogenerated electron–hole pairs, and the more radiative recombination occurs, and the higher PL intensity will be received (Feng et al., 2021; Li et al., 2021). Thus, it is obvious that the radiative recombination rate of samples with lower PL intensity is slower than that of pure Cu_2_MoS_4_ because the MXene with high electron conductivity can separate and transfer electrons from photogenerated exciton which prevents the radiative recombination and increases the concentration of carriers. Moreover, the separated carriers can efficiently facilitate crystal lattice vibrations after interacting with hot carriers produced by MXene because of the NIR-induced LSPR effect, thus leading to elevated temperatures (Zhou et al., 2020; Wang et al., 2021). However, when the ratio of Cu_2_MoS_4_ is excessive, the PL intensity becomes stronger and more radiative recombination occurs, which could be caused by aggregation issues and may weaken the photothermal performance.

**FIGURE 3:**
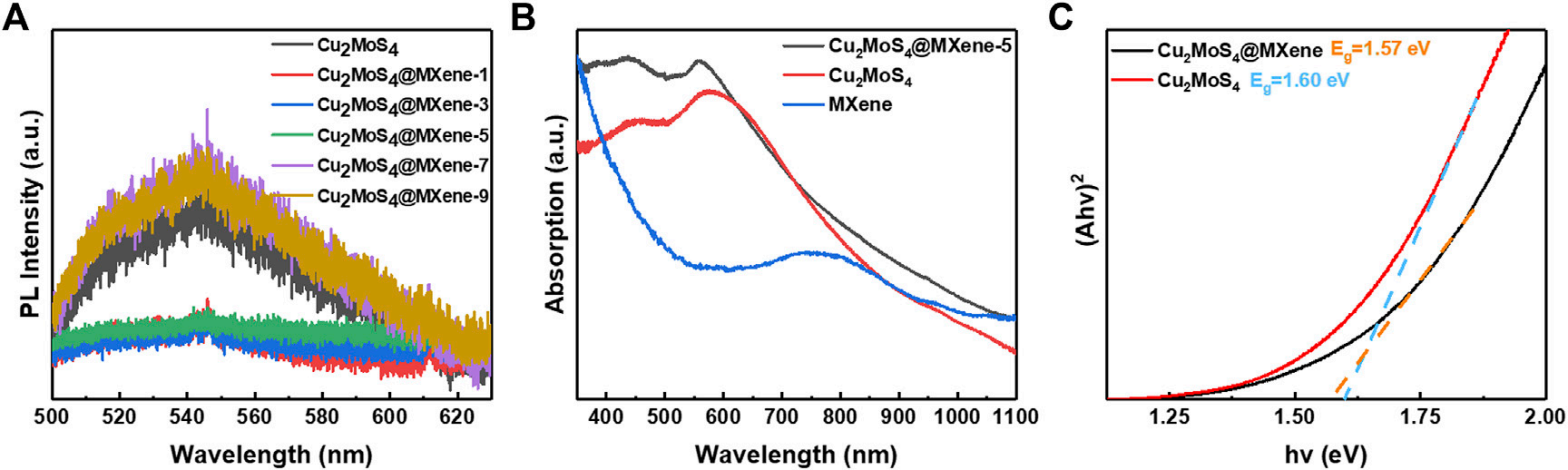
Optical properties of Cu_2_MoS_4_@MXene nanocomposites. **(A)** PL spectra (excited by a 325-nm laser from 500 to 630 nm) of Cu_2_MoS_4_ and Cu_2_MoS_4_@MXene nanocomposites. **(B)** UV-vis-NIR absorption spectra of Cu_2_MoS_4_, Cu_2_MoS_4_@MXene, and MXene. **(C)** Tauc plots of Cu_2_MoS_4_ and Cu_2_MoS_4_@MXene.

Figure 3B is the UV-vis-NIR absorption spectrum of Cu_2_MoS_4_ and Cu_2_MoS_4_@MXene nanocomposites, and the pattern illustrates that the nanocomposites exhibit a stronger absorption than the pure Cu_2_MoS_4_ in a nearly full band spectrum, which is another cause of high photothermal performance. As shown in Figure 3B, the absorption spectra of pure MXene shows a weak absorption intensity in visible and near-infrared region compared with Cu_2_MoS_4_@MXene nanocomposite. Furthermore, according to Tauc’s formulation (Wood and Tauc, 1972; Tauc, 1974), the optical band gaps can be calculated as around 1.60 and 1.57 eV for pure Cu_2_MoS_4_ and Cu_2_MoS_4_@MXene nanocomposites, respectively (Figure 3C). The narrowing band gap can be explained by the band theory that when the high work function MXene nanosheets come in contact with Cu_2_MoS_4_, the electrons will be drawn from a higher Fermi level of Cu_2_MoS_4_ to MXene, and when this process achieves a balance, the Fermi level of Cu_2_MoS_4_ will be brought down. So the band gap of nanocomposites is narrower than that of pure Cu_2_MoS_4_. In addition, this band gap engineering can promote the separation of photogenerated exciton and improve the photothermal performance, thus conforming to the result of the PL spectrum mentioned previously.

### Photothermal Performance

**FIGURE 4:**
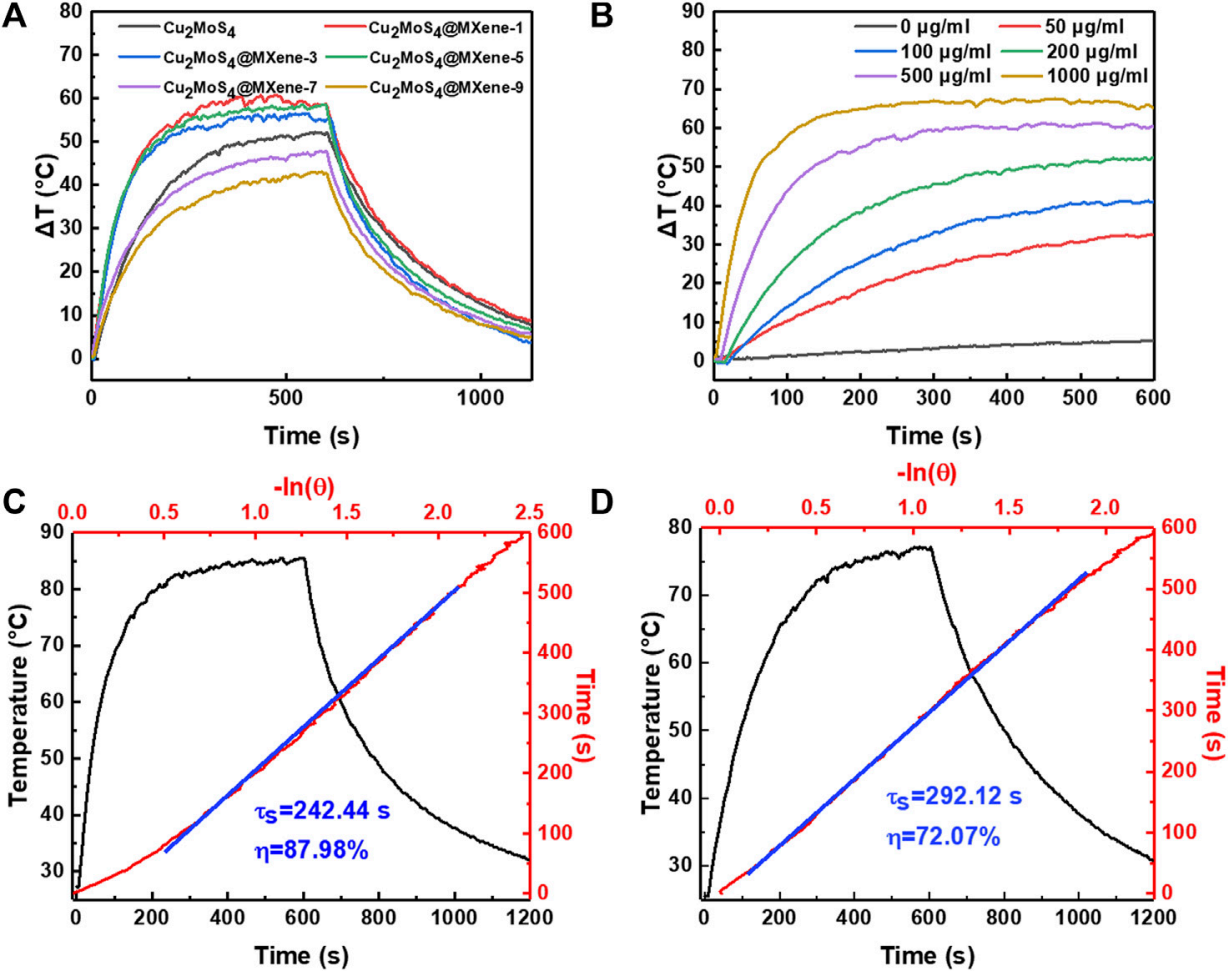
Photothermal performance of Cu_2_MoS_4_ and Cu_2_MoS_4_@MXene. **(A)** Photothermal activity of Cu_2_MoS_4_ and Cu_2_MoS_4_@MXene nanocomposites. **(B)** Concentration-dependent temperature change curves of Cu_2_MoS_4_@MXene-5. **(C,D)** Heating–cooling curves and linear time constant curves of Cu_2_MoS_4_@MXene and Cu_2_MoS_4_.

On account of the previously mentioned results, we forecast that the high-quality Cu_2_MoS_4_@MXene nanocomposites possess better photothermal performance than pure Cu_2_MoS_4_ due to the novel effects caused by the composite process, e.g., band engineering, electron transfer regulatory effect, and anchored effect. Thus, the NIR thermal conversion performance of Cu_2_MoS_4_ and Cu_2_MoS_4_@MXene nanocomposites was tested to verify this forecast. As shown in Figure 4A, under NIR light (1.0 W/cm^2^ at 808 nm) for 10 min, the temperature of each sample (500 μg/ml) has an obvious increase, and the highest ΔT can reach more than 55°C with two samples, i.e., Cu_2_MoS_4_@MXene-1 and Cu_2_MoS_4_@MXene-5, which is higher than pure Cu_2_MoS_4_ (50°C). However, the photothermal performance of samples marked as Cu_2_MoS_4_@MXene-7 and Cu_2_MoS_4_@MXene-9 is worse, which may be caused by the aggregation issue mentioned earlier. In addition, the temperature change is also dependent on concentration and laser power density which further demonstrates the distinguished photothermal conversion property of Cu_2_MoS_4_@MXene nanocomposites (Figure 4B and Supplementary Figure S4) (Hao et al., 2021). Taking into account the photostability of Cu2MoS4@MXene-1 and Cu2MoS4@MXene-5 (Supplementary Figure S5), Cu2MoS4@MXene-5 has the best performance, which matches the microscopic morphology and optical properties. When compared with the corresponding NIR thermal time constants (τ_s_) and conversion efficiency (*η*) of pure Cu_2_MoS_4_ (292.12 s and 72.07%), the better τ_s_ and η of Cu_2_MoS_4_@MXene-5 are calculated as 242.44 s and 87.98%, respectively (Figure 4C and Figure 4D) (Chang et al., 2019; Chen et al., 2019; Li et al., 2021). Therefore, based on all of these experimental data, it is evident that the composite process efficiently promoted the photothermal conversion ability.

### Mechanism of Photothermal Performance

To reveal the reasons for the enhanced performance of Cu_2_MoS_4_@MXene nanocomposites, their photothermal mechanism is proposed based on the previously analyzed experimental data. As reported in other previous work, under NIR irradiation, the electron in the photogenerated exciton will transit to the conduction band; then, the major electron in the excited state will recombine with the hole through radiating fluorescence; meanwhile, only a minority of electron–hole pairs have a photothermal effect due to nonradiative recombination, which can be described as the photothermal mechanism (Li et al., 2021). However, when the high work function (5.28 ± 0.03 eV) MXene is introduced, the heterojunction formed between Cu_2_MoS_4_ and MXene should be regarded as the transfer barrier (Regulacio et al., 2018; Lin et al., 2019; Yang et al., 2019; Prabaswara et al., 2020; Li et al., 2021). Referring to the band theory, the excited electron will irreversibly migrate from the Cu_2_MoS_4_ to MXene until their Fermi level reaches equilibrium; then, this migration can be accelerated because of the high-electron conductivity of MXene, and this process is defined as the band engineering caused by electron transfer regulatory effect. Obviously, the major excited electron of Cu_2_MoS_4_ in Cu_2_MoS_4_@MXene nanocomposites will migrate to MXene instead of radiatively recombining with the hole, so the probability of nonradiative recombination can multiply which improves the photothermal performance (Li et al., 2021).

Meanwhile, the UV-vis spectrum and Raman spectrum indicate an LSPR effect of MXene at 800 nm (1.5 eV) which can be attributed to an out-of-plane transverse plasmonic resonance, and owing to the LSPR effect, the MXene can generate the hot carriers under vis-NIR irradiation. Then, the injected hot carriers can further assist the separation and transfer of electrons and prevent the radiative recombination which is another crucial reason for improved photothermal performance.

In brief, after the hydrothermal process, Cu_2_MoS_4_ was anchored on the surface of MXene nanosheets, and the electron spontaneously migrates across the transfer barrier (electron transfer regulatory effect and band engineering). Then, with the synergy of MXene’s LSPR effect, the nonradiative recombination, i.e., the performance of photothermal conversion, can be efficiently accelerated.

## Conclusion

In summary, in order to improve the photothermal performance of Cu_2_MoS_4_, the MXene nanosheets were introduced, and the Cu_2_MoS_4_@MXene nanocomposite was successfully synthesized. Due to the superior electron conductivity and high work function of MXene, the motion of the electron was changed at the heterostructure of Cu_2_MoS_4_ and MXene, and the electron can migrate from Cu_2_MoS_4_ to MXene which promotes the nonradiative recombination and generates heat. Also, the experimental results show that the radiative combination was evidently prevented, indicating an accelerated nonradiative combination, and the enhanced photothermal conversion efficiency of Cu_2_MoS_4_@MXene nanocomposite can reach 87.98% compared with the pure Cu_2_MoS_4_ (*η* = 72.07%). Then, a mechanism was proposed based on the electron transfer regulatory effect and LSPR effect. Finally, this work provides an efficient method to enhance the photothermal performance of phototherapy reagents and make them play a great role in cancer treatment.

## Data Availability

The original contributions presented in the study are included in the article/Supplementary Material, further inquiries can be directed to the corresponding authors.
