# Supplementary material for: A Strategy to Design Cu2MoS4@MXene Composite With High Photothermal Conversion Efficiency Based on Electron Transfer Regulatory Effect
Source: Front Bioeng Biotechnol. 2022 May 13;10:902312. doi: 10.3389/fbioe.2022.902312 (PMC9136138; doi:10.3389/fbioe.2022.902312)
Supplement: Supplementary file 1 [file DataSheet1.docx]

Supplementary Material


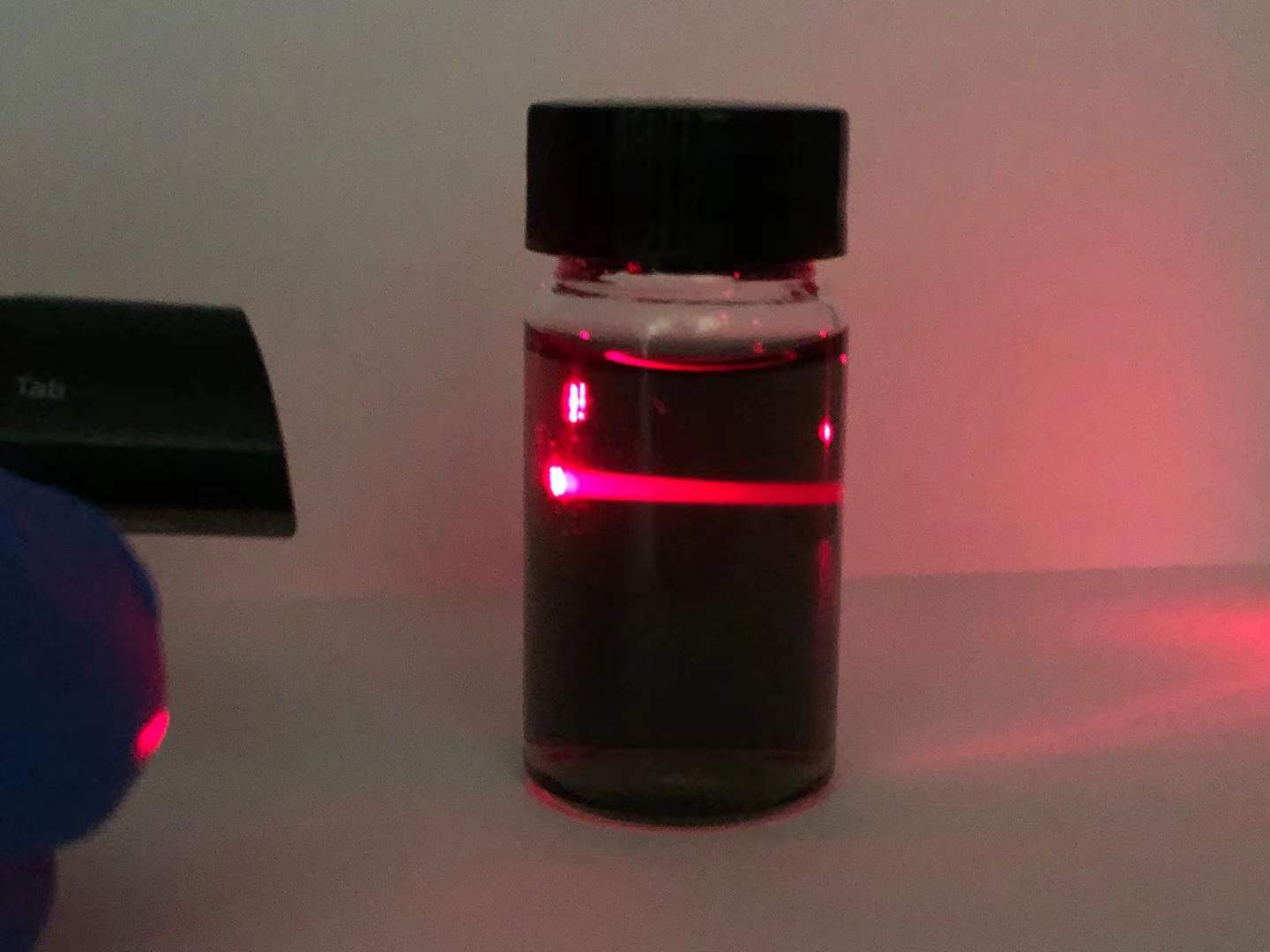


Figure S1. The Tyndall effect of MXene nanosheets colloidal aqueous solution.


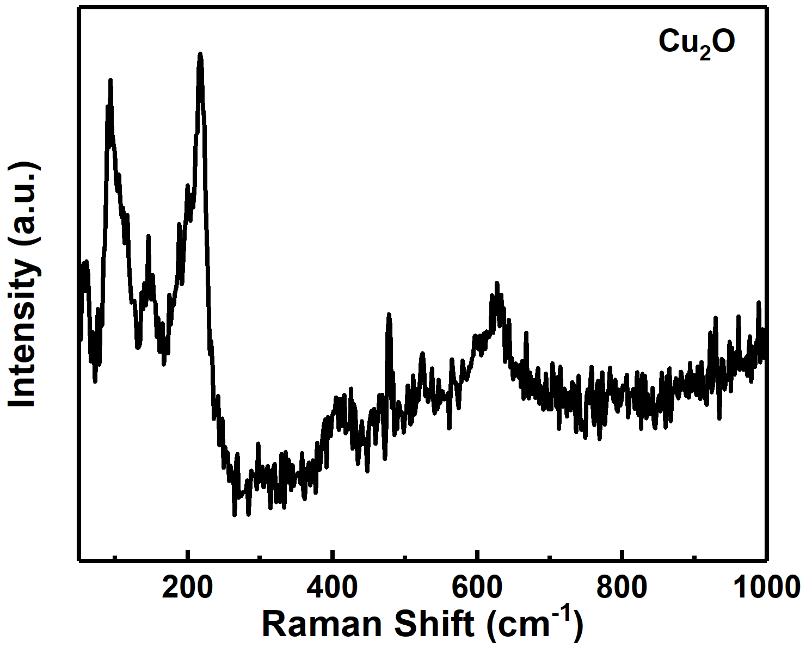


Figure S2. Raman spectra of Cu_2_O precursor.


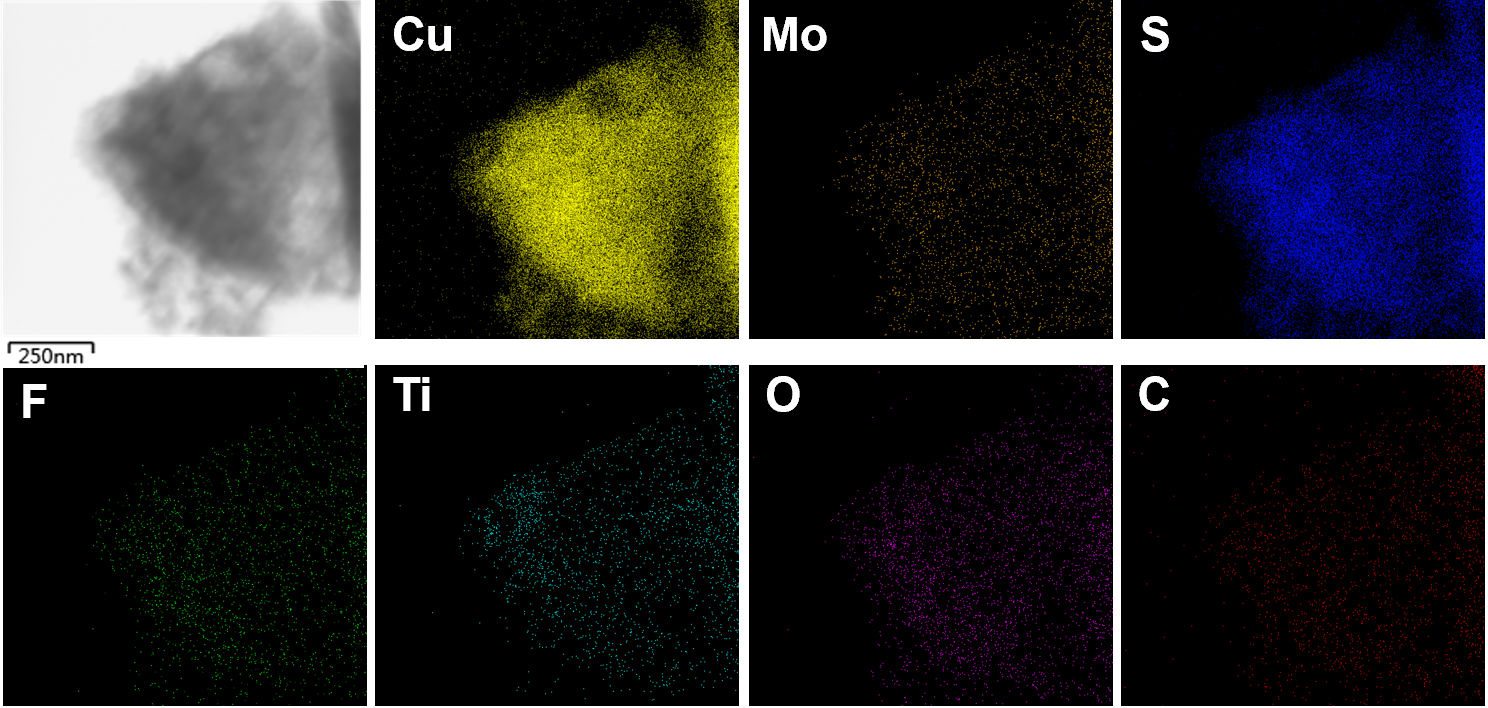
 Figure S3. EDS mapping of Cu_2_MoS_4_@MXene-9.


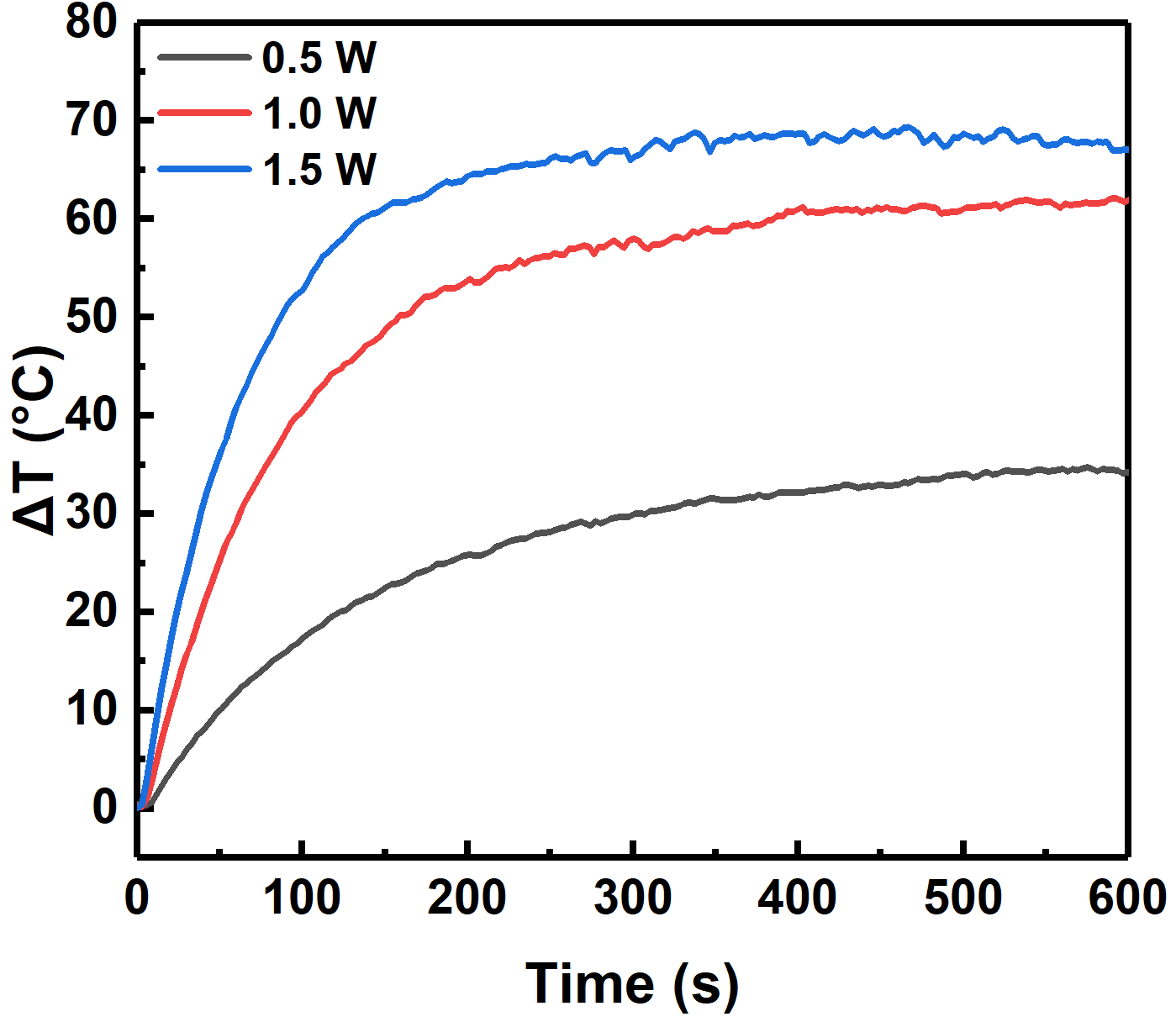


Figure S4. Laser power-dependent temperature change curves of Cu_2_MoS_4_@MXene-5.


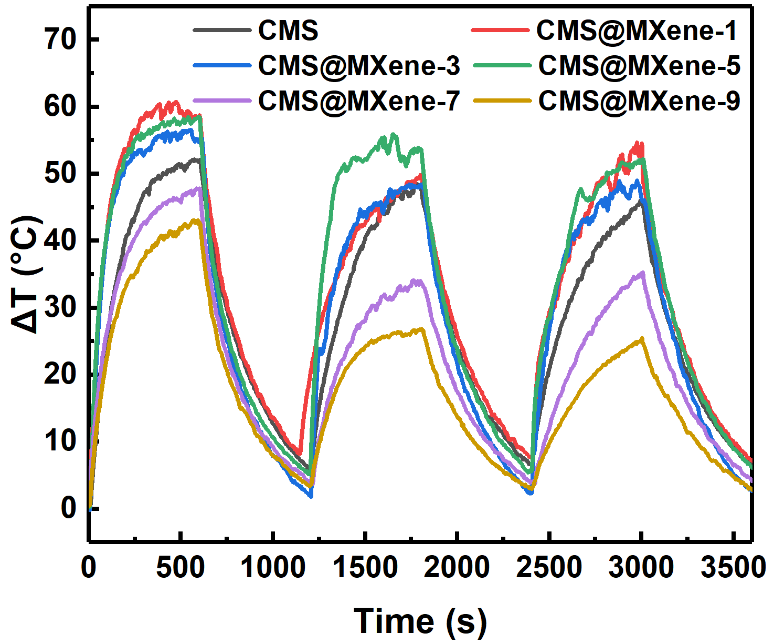


**Figure S5**. Photothermal stability of Cu_2_MoS_4_ and Cu_2_MoS_4_@MXene nanocomposites.
